# Comparing transcranial magnetic stimulation and esketamine treatment response trajectories in resistant depression

**DOI:** 10.1016/j.jad.2026.122107

**Published:** 2026-06-07

**Authors:** Lindsay L. Benster, Jordan N. Kohn, Benjamin Wade, Noah Stapper, Cory R. Weissman, Jean-Philippe Miron, Zafiris J. Daskalakis, Lawrence G. Appelbaum

**Affiliations:** a Department of Psychiatry, University of California San Diego, La Jolla, CA, USA; b Herbert Wertheim School of Public Health and Human Longevity Science, University of California San Diego, La Jolla, CA, USA; c Department of Psychiatry, Massachusetts General Hospital and Harvard Medical School, Boston, MA, USA

**Keywords:** rTMS, Esketamine, Treatment-resistant depression

## Abstract

**Objective:**

Repetitive transcranial magnetic stimulation (rTMS) and intranasal esketamine are FDA-approved for treatment-resistant depression (TRD), yet comparative real-world data on response trajectories and predictors of outcomes remain limited.

**Methods:**

A retrospective analysis was performed using electronic medical records from UC San Diego Health. Adults with TRD treated with rTMS (*n* = 279) or intranasal esketamine (*n* = 93) between 2017 and 2025 were included. The primary outcome was clinical response (≥50% PHQ-9 reduction). Time-to-response was assessed using inverse probability treatment weighted (IPTW) Cox models; Kaplan-Meier curves and log-rank tests provided descriptive comparisons. Covariates included age, trauma history, anxiety comorbidity, benzodiazepine use, tobacco use history, BMI, and baseline symptom severity. Secondary outcomes included remission (PHQ-9 < 5) and suicidal ideation (SI).

**Results:**

Esketamine demonstrated earlier response over 90 days (RMST difference = −11.94 days) and faster time-to-response in the IPTW Cox model (HR = 1.62, *p* = 0.005); KM estimates showed median response at 36 vs. 49 days (*p* = 0.0096) with convergence by ~90 days. Cumulative response and remission rates were numerically higher for esketamine (68.8% / 45.2%) than rTMS (59.4% / 40.1%), supporting a speed-of-response difference rather than superior overall efficacy. SI improved more rapidly with esketamine (median 9 vs. 26 days; *p* = 0.001). In the rTMS cohort, comorbid anxiety (HR = 0.69, *p* = 0.039) and benzodiazepine use (HR = 0.73, *p* = 0.046) predicted slower response, while former tobacco use predicted faster response (HR = 1.31, *p* = 0.006). No significant predictors emerged for esketamine.

**Conclusions:**

Esketamine was associated with earlier observed antidepressant and anti-suicidal improvement than rTMS. Baseline factors, including benzodiazepine use, may help inform expectations regarding rTMS response trajectory.

## 1. Introduction

Treatment-resistant depression (TRD) is a debilitating condition characterized by inadequate response to two or more antidepressant trials administered at appropriate doses and durations (Fava, 2003; Rush et al., 2006). Patients with TRD experience persistent depressive symptoms, elevated risks of medical comorbidities, and increased healthcare utilization, underscoring the urgent need for effective therapies (Berlim and Turecki, 2007). Repetitive transcranial magnetic stimulation (rTMS) and ketamine have garnered considerable attention in TRD. rTMS is a non-invasive neuromodulation technique utilizing magnetic fields to stimulate mood-relevant neural circuits (Downar and Daskalakis, 2013). Randomized controlled trials (RCTs) demonstrated that rTMS can produce clinically meaningful antidepressant effects in TRD, with a favorable safety profile and minimal side effects (George et al., 2013; O’Reardon et al., 2007). Ketamine is a rapid-acting antidepressant, offering clinical benefits within hours of administration (Coyle and Laws, 2015). Esketamine is the S-enantiomer of ketamine and most commonly administered intranasally. Unlike conventional antidepressants that target monoamine signaling, esketamine acts primarily on the glutamatergic system, modulating synaptic plasticity and neuronal connectivity (Appelbaum et al., 2023). Its rapid therapeutic onset makes it particularly promising for TRD, especially those with imminent risk of self-harm (Murrough et al., 2013; Zarate et al., 2006).

Despite robust evidence supporting the efficacy of rTMS and esketamine, their comparative response rates and treatment dynamics in routine clinical practice remain unclear. Further, guidance on optimal treatment selection is lacking, as RCTs have largely evaluated each modality in isolation (Murrough et al., 2013; O’Reardon et al., 2007; Zarate et al., 2006). Furthermore, RCT efficacy estimates may not translate into real-world effectiveness given patient heterogeneity and clinical complexities in treatment-seeking populations. Consequently, clinicians face limited guidance for treatment selection and timing, leaving a critical gap in understanding how these interventions compare in everyday practice (Berlim and Turecki, 2007; Fava, 2003).

Esketamine and rTMS differ in treatment cadence for logistical, safety, and evidence-based effectiveness reasons. Intranasal esketamine is delivered in a supervised clinic setting with post-dose monitoring and often follows an induction schedule of twice-weekly dosing for 4 weeks followed by weekly or every-other-week maintenance. rTMS is typically administered as brief weekday sessions over ~4–6 weeks (≈20–30+ sessions), consistent with pivotal trials (O’Reardon et al., 2007) and consensus recommendations. Respective FDA-approved protocols are informed by extant literature, delivery requirements, and reimbursable course structures.

This study leverages electronic medical records (EMR) of patients with TRD treated by UC San Diego Health Interventional Psychiatry Program (UCSD-IPP). Harnessing real-world data enables evaluation of response rates, time-to-response, and symptom trajectories, across a heterogeneous patient population, facilitating examination of broader patient characteristics and enhancing clinical relevance (Rush et al., 2006). This approach investigates the following questions: (1) do rTMS and esketamine differ in time-to-response and overall outcomes in TRD; (2) which clinical factors predict response to rTMS and esketamine; and (3) do rTMS and esketamine differ in time-to-response and outcomes in suicidal ideation (SI)?

By characterizing response patterns and temporal profiles, clinicians may better use these therapies in real time for emergent needs and prospectively to predict which patients are more likely to benefit from each treatment, thereby optimizing intervention selection and reducing trial-and-error.

## 2. Methods

### 2.1. Participants

Participants (*n* = 372) were adults (≥18 years) with TRD who initiated rTMS (*n* = 279) or intranasal esketamine (*n* = 93) through UCSD-IPP and identified through Epic medical records (Verona, WI, USA). Inclusion criteria consisted of a primary diagnosis of Major Depressive Disorder (MDD) meeting DSM-5 criteria and EMR-documented history of insufficient response to ≥2 antidepressant trials. Individuals with psychiatric comorbidities documented in their EMR (problem list and encounter diagnoses) were included. Demographic and clinical variables including age, gender, body mass index (BMI), psychiatric comorbidities, trauma history, benzodiazepine use, and concurrent substance use (including alcohol, cannabis, and illicit substances) were systematically recorded. These were selected based on prior work and the extant literature (Benster et al., 2025; Chou et al., 2021; Garcia et al., 2025).

### 2.2. Treatment protocols

Comprehensive details of protocol type, coil localization, motor-threshold determination, and stimulation parameters for all rTMS protocols have been published previously (Benster et al., 2025). A brief overview is provided here: rTMS was delivered using standard MagVenture (MagVenture A/S, Farum, Denmark) and BrainsWay (BrainsWay Ltd., Jerusalem, Israel) clinical protocols, including intermittent theta-burst stimulation (iTBS), bilateral theta-burst stimulation (BiTBS), high-frequency left dorsolateral prefrontal cortex (DLPFC), and low-frequency right DLPFC stimulation. These protocols were pooled for the present comparative analyses to reflect real-world clinical rTMS delivery, and protocol selection reflected routine clinical decision-making informed by patient presentation and treatment history. rTMS sessions were typically conducted multiple times per week, with a standard treatment series comprising approximately 36 sessions, but varying widely (range 5–82 sessions; mean 38.45, SD =10.98).

**Fig. 1.**
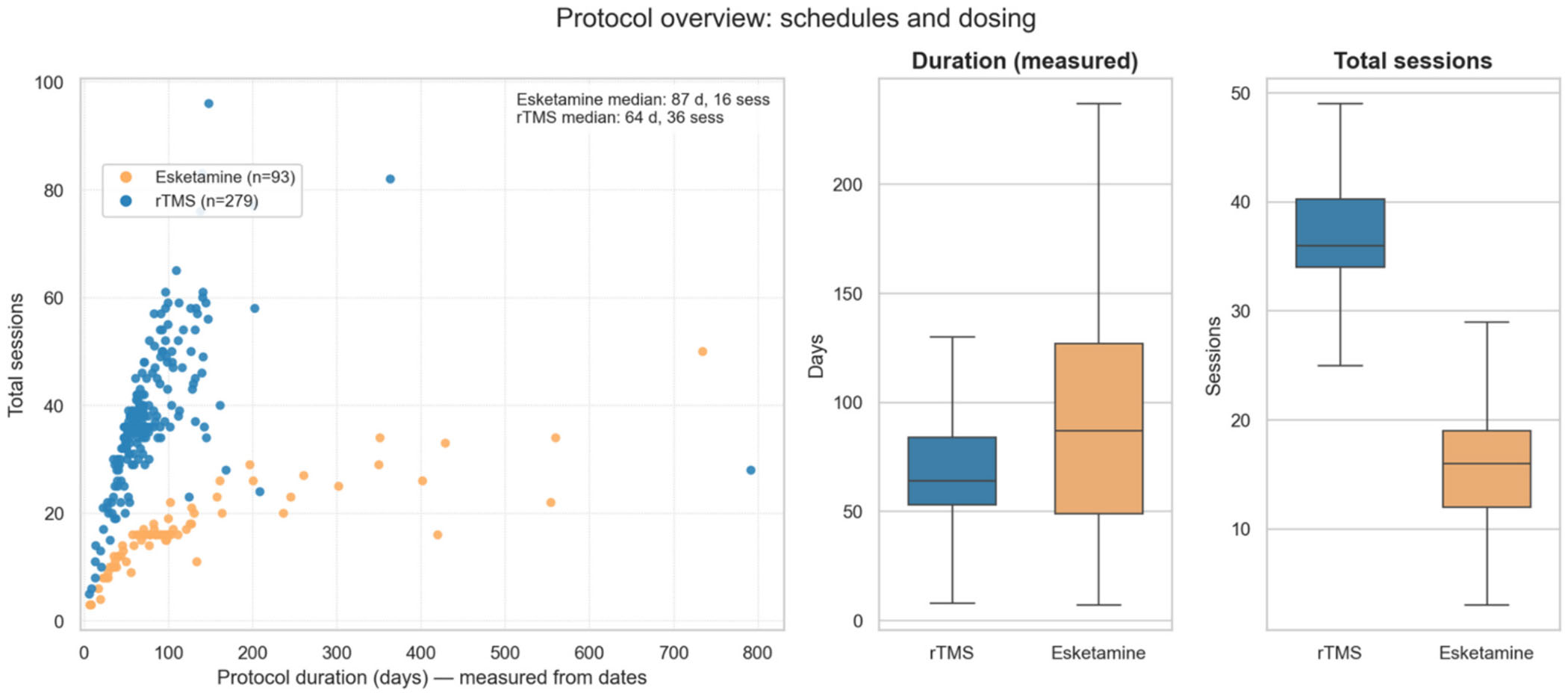
Protocol overview. (Left) Each point represents one patient, plotted by protocol duration (days from baseline to final session) versus total sessions (clinic-recorded totals) by treatment group (blue: rTMS; orange: esketamine). Boxplots of days (center) and sessions (right) summarize the distributions of protocol duration and total sessions by treatment group. Outliers are omitted from the boxplots for visual clarity. **Alt text:** Scatter and boxplots show esketamine had longer protocol duration but fewer sessions than rTMS, with broader duration variability. (For interpretation of the references to colour in this figure legend, the reader is referred to the web version of this article.)

Esketamine (SPRAVATO; Janssen Pharmaceuticals, Titusville, NJ, USA) was administered intranasally under clinical supervision, typically twice weekly. Patients initiated at 56 mg with escalation to 84 mg if 56 mg was tolerated. Dosage and session frequency were individualized based on clinician judgment, tolerability, and scheduling constraints. A minimum of 5 rTMS and 3 esketamine sessions was required for inclusion to ensure sufficient exposure for an interpretable clinical trajectory. Discontinuation before these thresholds generally reflected inadequate follow-up time or early tolerability issues rather than meaningful treatment engagement, precluding valid estimation of time-to-response. Discontinuation after meeting minimum thresholds was handled through right-censoring (a standard survival-analysis approach in which individuals not responding by their last visit were treated as having unknown response times beyond that point), allowing patients who stopped treatment prematurely to contribute follow-up time without being misclassified as responders. Treatment frequency and intersession interval plots are available in Fig. 1 and the Supplementary Materials.

### 2.3. Clinical outcome measures

The primary outcome was depression treatment response, measured by the Patient Health Questionnaire-9 (PHQ-9) total score. The PHQ-9 is a self-administered, nine-item questionnaire designed to evaluate the severity of depressive symptoms as defined by the DSM-5 criteria for a Major Depressive Episode (MDE). Total scores range from 0 to 27 with established severity thresholds. PHQ-9 assessments were collected pre-treatment and longitudinally (at varying intervals for rTMS; at each session for esketamine). Response was defined as ≥50% reduction from baseline; remission as a PHQ-9 score < 5 post-treatment and examined descriptively. Additionally, PHQ-9 item 9 was used to assess SI, with improvement operationalized as a ≥ 1-point reduction from an initial score greater than zero (Stapper et al., 2025).

### 2.4. Statistical analysis

Analyses were conducted in Python (v3.9.12) using *scikit-learn* and *lifelines*. The primary analytic strategy implemented doubly robust estimation using an inverse probability of treatment-weighted (IPTW) Cox proportional hazards (CPH) model. Propensity-score (PS) weights were applied to create a pseudo-population with balanced baseline covariates between groups and the treatment effect was estimated in that weighted sample. PS for receiving esketamine (versus rTMS) were estimated by logistic regression using baseline covariates: age, baseline PHQ-9, BMI, anxiety-disorder comorbidity, psychological-trauma history, and former-tobacco use (Supplemental Table S5). Stabilized IPTW were computed as:

$$w_{i}=Z_{i}\frac{p_{t}}{\hat{ps_{i}}}+\left(1-Z_{i}\right)\frac{1-p_{t}}{1-\hat{ps_{i}}}$$

where *Z_i_* denotes treatment assignment, $\hat{ps_{i}}$ is the estimated PS, and *p_t_* is the sample treatment prevalence (*p_t_* = 0.245). Weights were winsorized at 1st and 99th percentiles to reduce variance from extreme values. Covariate balance was evaluated using standardized mean differences before and after weighting, with absolute standardized mean differences <0.10 considered acceptable; weight distributions and effective sample size were inspected to assess IPTW adequacy. As a sensitivity analysis, an unweighted penalized CPH including treatment and the same baseline covariates used in the PS model was fit; ridge regularization was selected by cross-validation (Supplementary Methods S1). To address potential confounding by treatment history, prior rTMS exposure was added to the propensity-score model in an additional IPTW sensitivity analysis; a second sensitivity analysis excluded all patients with prior rTMS exposure. To address potential ascertainment bias from differential PHQ-9 assessment frequency, time-to-response was recalculated using harmonized 7-day and 14-day assessment windows, assigning first response to the end of the interval in which it was observed. Within-arm CPH models evaluated predictors of time-to-response within each modality (age, trauma history, benzodiazepine use, concurrent substance use, BMI, and number of psychiatric comorbidities). Proportionalhazards assumptions were assessed with Schoenfeld residuals (variable-wise and global tests), and multicollinearity was evaluated using variance inflation factor (VIF; VIF < 5 considered acceptable), all of which were < 5. Variable-wise Schoenfeld testing indicated potential nonproportionality for the treatment covariate (*p* = 0.029; Supplemental Table S6); restricted mean survival time (RMST; 0–90 days) was therefore computed to provide an interpretable timescale estimate less dependent on the proportional-hazards assumption. Logistic regression was used to model sustained endpoint response (response at treatment completion) with the same predictor set, plus concomitant benzodiazepine use, concurrent substance use (alcohol, tobacco, cannabis, or illicit substances), and number of psychiatric comorbidities, informed by existing literature and from analyses conducted on a smaller subset of the rTMS sample of this database. All hypothesis tests were two-sided with α = 0.05.

As secondary descriptive analyses, unadjusted Kaplan-Meier (KM) curves compared time-to-response (days since treatment initiation) between rTMS and esketamine; response curves were plotted as Kaplan-Meier-derived cumulative response probability, calculated as 1 minus the KM survival estimate. Time-to-event was computed in whole days from the baseline PHQ-9 assessment date; because EHR symptom assessments were not uniformly available at sub-day timestamp resolution, events occurring within 24 h of baseline could be recorded as 0 days. Cumulative response probabilities at multiple predefined time points were calculated. Log-rank tests were employed to compare survival curves between groups. Patients not achieving response by their last attended session were right-censored at that session date, removing them from the risk set without registering as events. Only true response events (ΔPHQ-9 ≥ 50%) generated step increases in the KM curves; these events were recorded even if treatment continued after achieving response. Number-at-risk details are provided in Section 3.3. A parallel KM analysis was conducted for SI trajectories; patients with a baseline SI score of zero (i.e., no reported SI) were excluded, yielding an SI subsample of rTMS *n* = 147 and esketamine *n* = 55.

## 3. Results

### 3.1. Descriptive statistics

Baseline demographic and clinical characteristics are detailed in Table 1 and the Supplementary Materials. No between-group differences were observed in age, BMI, anxiety comorbidity, concurrent substance use, former tobacco use, prior ECT, or baseline PHQ-9. Trauma History significantly differed, with a higher prevalence in the esketamine (63.8%) versus the rTMS group (46.3%). Prior rTMS treatment was also more common in the esketamine group (47%) than the rTMS group (11.1%). Data on prior esketamine use were unavailable. Session counts ranged from 5 to 82 for rTMS (mean = 38.45; SD = 10.98; median = 36), and 3–44 sessions for esketamine (mean = 10.6; SD = 6.5; median = 10). Additional session and follow-up metrics are provided in Supplemental Table S3.

Overall response rates at any point during treatment were 59.43% for rTMS and 68.82% for esketamine. Remission rates were 40.14% and 45.16%, respectively. End-of-treatment response occurred in 45% of rTMS patients and 51% of esketamine patients.

### 3.2. Between-group time-to-response analysis

A total of 353 patients had sufficient data for inclusion in the time-to-response analysis; 219 patients met response criterion and 134 were right-censored at their last contact. After balancing baseline differences between groups, esketamine was associated with earlier average response over the 90-day horizon (RMST difference = −11.94 days [esketamine – rTMS]). The IPTW Cox model similarly indicated faster time-to-response for esketamine (HR = 1.62, 95% CI 1.16–2.26, *p* = 0.005). Because the treatment covariate showed evidence of nonproportionality, the HR was interpreted as a summary of the observed time-to-response difference rather than a constant treatment effect over follow-up. IPTW improved covariate balance across propensity-score covariates, reducing the maximum absolute standardized mean difference from 0.381 before weighting to 0.032 after weighting; no weighted covariates had absolute standardized mean differences >0.10. Winsorized stabilized weights ranged from 0.573 to 2.089, with an effective sample size of 353 (Supplementary Table S8). Proportional-hazards testing indicated evidence of nonproportionality for the treatment variable (χ^2^(1) = 4.77, *p* = 0.029), although the global test was not significant (χ^2^(9) = 8.65, *p* = 0.47). Similar findings were observed in the sensitivity analysis (HR = 1.23, 95% CI 1.04–1.45, *p* = 0.014; Supplementary Table S7). Results were consistent in a sensitivity analysis aligning minimum treatment exposure across modalities by excluding rTMS patients with <12 sessions (rTMS *n* = 274; esketamine *n* = 93; IPTW HR = 1.65, 95% CI 1.19–2.31; covariate-adjusted HR = 1.52, 95% CI 1.16–2.00; RMST_0–90_d: −12.6 and – 9.8 days). Prior rTMS exposure was more common in the esketamine group than the rTMS group (47.3% [44/93] vs. 11.1% [31/279]). Findings remained robust after adding prior rTMS exposure to the propensity-score model (HR = 1.95, 95% CI 1.40–2.71, *p* = 0.0001). In a second sensitivity analysis, excluding all patients with prior rTMS exposure (*n* = 75) yielded an analytic sample of 297 patients (rTMS *n* = 248; esketamine *n* = 49), and the association between esketamine and faster time-to-response remained significant (HR = 1.89, 95% CI 1.24–2.89, *p* = 0.0029). Sensitivity analyses using harmonized assessment windows yielded consistent results for both 7-day windows (HR = 1.54, 95% CI 1.11–2.12, *p* = 0.0093) and 14-day windows (HR = 1.49, 95% CI 1.09–2.06, *p* = 0.0138).

### 3.3. Time-to-response for depression symptoms: rTMS versus esketamine

**Fig. 2.**
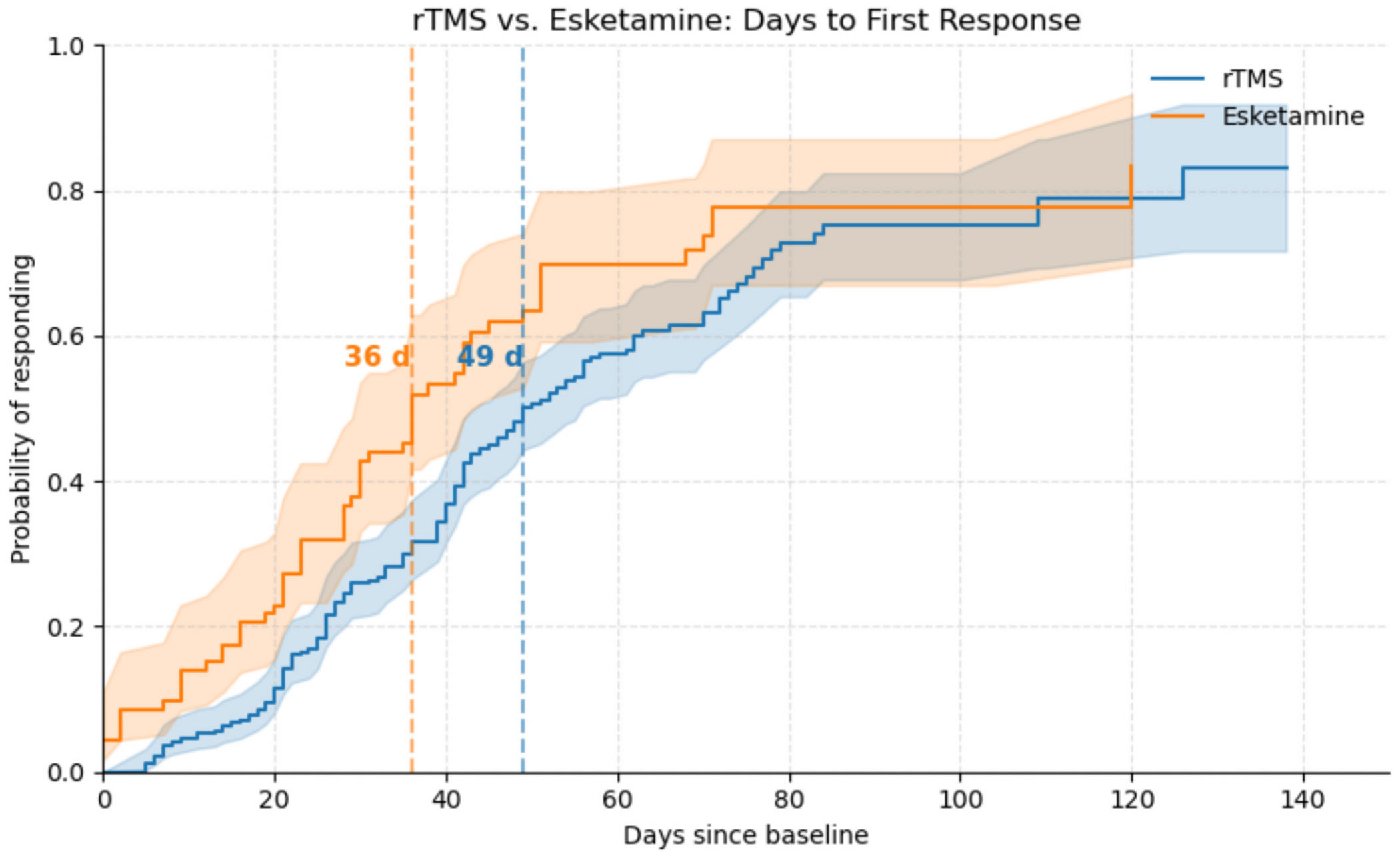
KM-derived cumulative response probability for rTMS and esketamine. Curves show cumulative response probability, calculated as 1 minus the Kaplan-Meier survival estimate, plotted against days since baseline for rTMS (blue) and esketamine (orange). The y-axis represents the probability of achieving response; shaded ribbons denote 95% confidence intervals. Vertical dashed lines mark the day on which each cohort first reached 50% cumulative response probability (rTMS: 49 days; esketamine: 36 days). Because censoring removes patients from the risk set over time, later increases reflect observed response events among a progressively smaller at-risk pool. **Alt text:** Response curves separate early, with esketamine improving faster than rTMS, but both groups converge by about 90 days. (For interpretation of the references to colour in this figure legend, the reader is referred to the web version of this article.)

As an unadjusted, descriptive complement, KM estimates were used to compare time-to-response between groups; Fig. 2 presents KM-derived cumulative response probability, calculated as 1 minus the KM survival estimate. Median time-to-response was 36 days for esketamine and 49 days for rTMS (log-rank test χ^2^ = 6.699, *p* = 0.0096). Cumulative response probabilities separated early, particularly in the first 30–60 days: at 5 days, 1.1% (rTMS) vs. 8.6% (esketamine); at 10 days, 4.6% (rTMS) vs. 14.0% (esketamine); at 20 days, 11.4% (rTMS) vs. 22.9% (esketamine); at 30 days, 26.0% (rTMS) vs. 42.6% (esketamine); at 60 days, 57.5% (rTMS) vs. 69.9% (esketamine); at 90 days, 75.3% (rTMS) vs. 77.8% (esketamine); they became similar by ~90 days, with minimal between-group differences thereafter through follow-up. Because KM estimates adjust for censoring, cumulative response probabilities exceed observed crude rates as the at-risk pool diminishes over time.

### 3.4. Clinical and demographic predictors of depression response

**Fig. 3.**
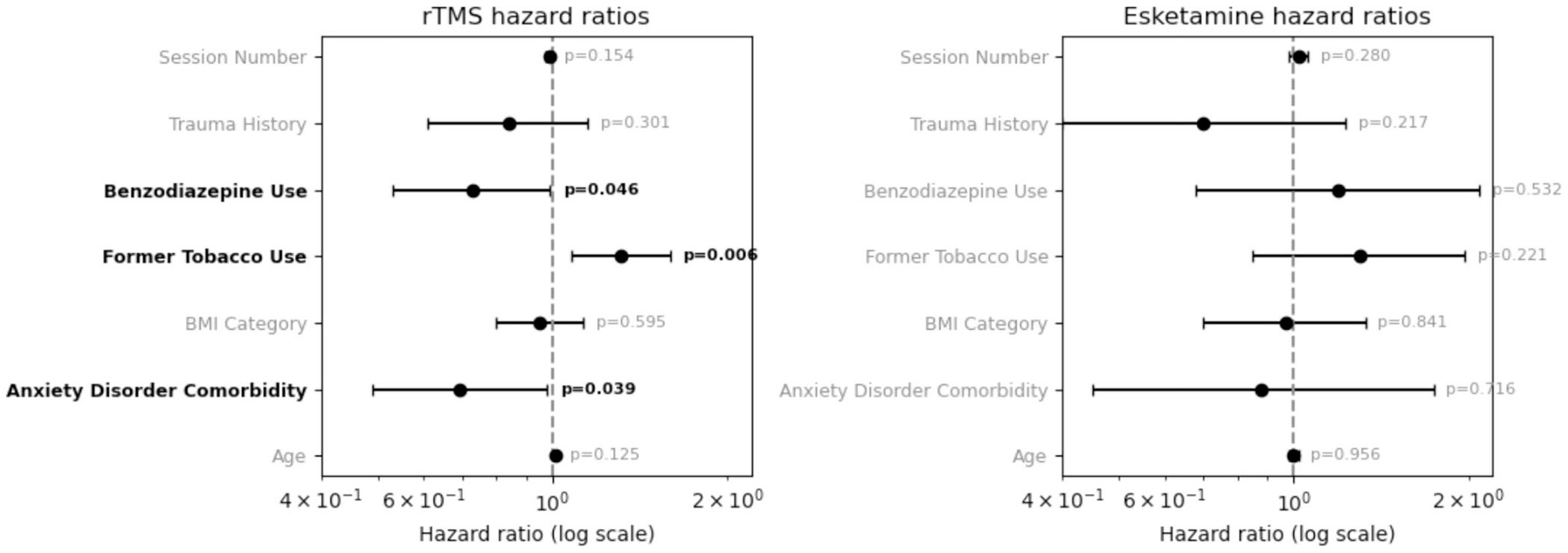
Hazard ratios for clinical response within rTMS and esketamine cohorts. Forest plots display hazard ratios and 95% confidence intervals for baseline predictors of time-to-response in rTMS (left) and esketamine (right). Values greater than 1 indicate faster response; values less than 1 indicate slower response. Significant predictors are shown in black and non-significant predictors in gray. **Alt text:** Forest plots show anxiety and benzodiazepine use predict slower rTMS response, while no significant predictors emerged for esketamine.

In the rTMS group, anxiety disorder comorbidity was associated with a 31% lower response hazard (HR = 0.69, 95% CI 0.49–0.98, *p* = 0.039), former tobacco use conferred a 31% higher hazard (HR = 1.31, 95% CI 1.08–1.59, *p* = 0.0059), and benzodiazepine usage predicted a 27% lower hazard (HR = 0.73, 95% CI 0.53–0.99, *p* = 0.0459). Age (HR = 1.01, 95% CI 1.00–1.02, *p* = 0.125), trauma history (HR = 0.84, 95% CI 0.61–1.15, *p* = 0.301), BMI (HR = 0.95, 95% CI 0.80–1.13, *p* = 0.595), and total sessions completed (HR = 0.99, 95% CI 0.97–1.00, *p* = 0.154) were not significant. In the esketamine group, no covariates reached significance. Coefficients, 95% CIs, and *p*-values are tabulated in Supplemental Table S1 and Fig. 3.

### 3.5. Time-to-SI improvement: rTMS versus esketamine

**Fig. 4.**
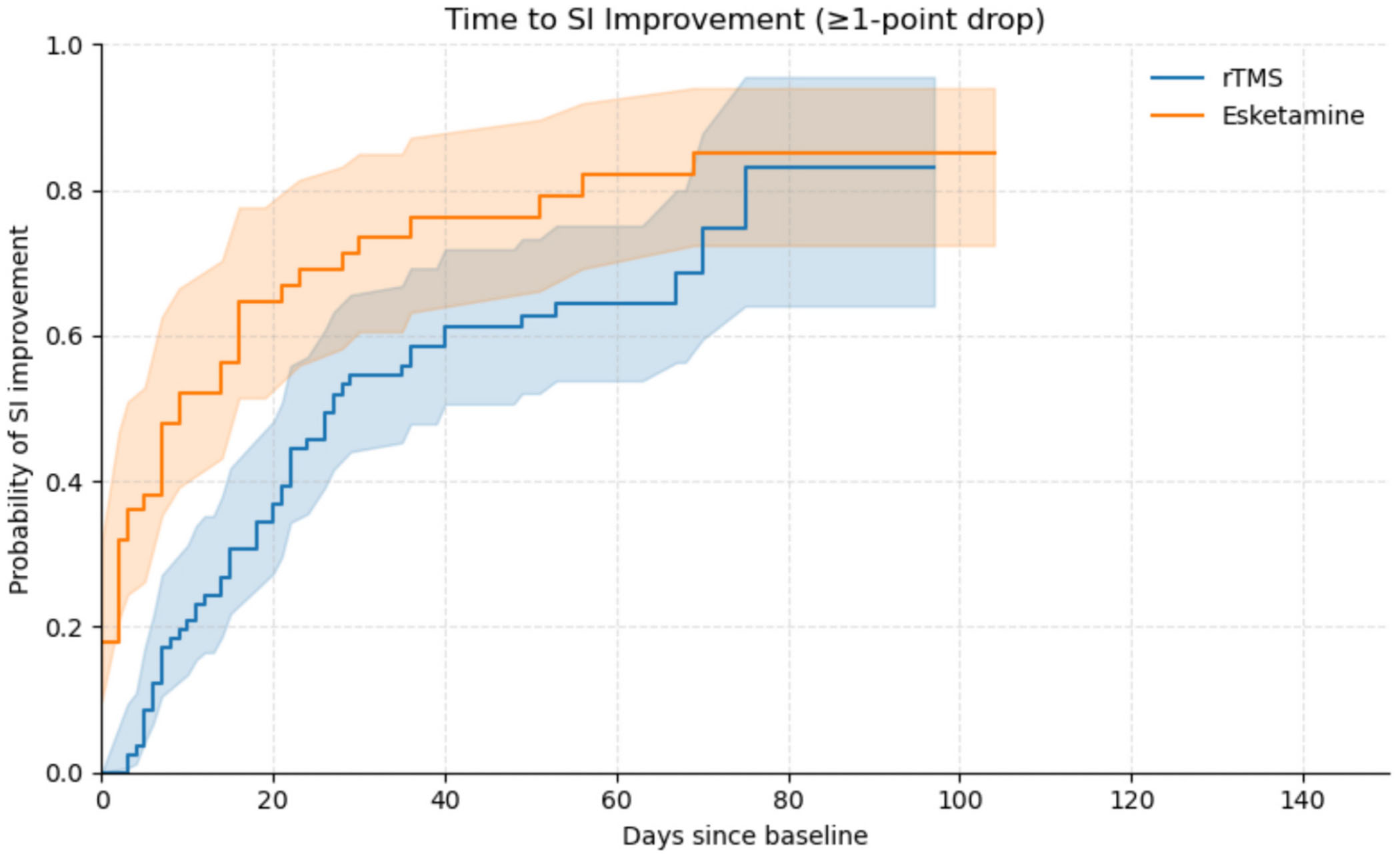
KM-derived cumulative probability for SI improvement. Curves show the KM-derived cumulative probability of SI improvement over days since baseline in rTMS (blue) and esketamine (orange). Higher values indicate greater probability of SI improvement over time. **Alt text:** Suicidality curves show faster early improvement with esketamine than rTMS, with differences narrowing over follow-up. (For interpretation of the references to colour in this figure legend, the reader is referred to the web version of this article.)

Median times to SI improvement were markedly shorter for the esketamine group (9 days) compared to the rTMS group (26 days; KM curve log-rank test: χ^2^ = 10.681, *p* = 0.0011). Cumulative probabilities of SI improvement were consistently higher for esketamine, indicating faster improvement: at 5 days, 9.7% (rTMS) vs. 40.0% (esketamine); at 10 days, 23.0% (rTMS) vs. 52.9% (esketamine); at 20 days, 39.5% (rTMS) vs. 66.1% (esketamine); at 30 days, 58.4% (rTMS) vs. 74.0% (esketamine); at 60 days, 67.5% (rTMS) vs. 81.6% (esketamine); at 90 days and beyond, probabilities stabilized around 83.0% (rTMS) and 84.7% (esketamine). SI improvement by timepoint is illustrated in Fig. 4 and Supplementary Table S2.

### 3.6. Clinical and demographic predictors of sustained response

Among patients who received rTMS, current benzodiazepine use was associated with lower odds of sustained response (OR = 0.49, 95% CI 0.28–0.88, *p* = 0.016), whereas concurrent substance use conferred higher odds (OR = 1.51, 95% CI 1.08–2.12, *p* = 0.017). No other covariates reached significance: age (OR = 1.01, 95% CI 0.99–1.03, *p* = 0.269), trauma history (OR = 1.26, 95% CI 0.75–2.11, *p* = 0.379), BMI (OR = 0.88, 95% CI 0.69–1.14, *p* = 0.344), number of comorbidities (OR = 0.89, 95% CI 0.72–1.12, *p* = 0.327), and baseline PHQ-9 severity (OR = 1.08, 95% CI 0.88–1.32, *p* = 0.470). The overall model showed modest discrimination (AUC = 0.696) and explanatory power (LRT: *p* = 0.0397; Pseudo-R^2^ = 0.0381). Within the esketamine-treated group, no predictors reached significance; baseline PHQ-9 severity showed a nonsignificant trend toward higher odds of response (OR = 1.24, 95% CI 0.98–1.57, *p* = 0.071). The esketamine model had higher discrimination (AUC = 0.747), but overall fit was not significant (LRT: *p* = 0.3525; Pseudo-R^2^ = 0.0624). Full model estimates are reported in Supplementary Table S4.

## 4. Discussion

This study compared real-world response trajectories for rTMS and esketamine in TRD, revealing esketamine was associated with faster observed antidepressant and anti-suicidal effects, while overall response estimates converged over longer follow-up. Thus, the primary distinction observed in this cohort was speed-of-response rather than clear superiority in overall effectiveness. Given evidence of nonproportionality for the treatment covariate, the hazard ratio should be interpreted as a summary of the observed time-to-response difference rather than a constant treatment effect over follow-up. The RMST analysis supported this pattern on the time scale, indicating earlier average response with esketamine over the 90-day horizon. While both were effective, esketamine yielded earlier symptom relief, whereas rTMS demonstrated a more gradual response trajectory and outcomes appeared more contingent on patient-specific factors. This timing difference remained consistent across sensitivity analyses, supporting the robustness of the observed trajectory while underscoring the need to interpret early separation in the context of treatment cadence, assessment timing, and clinical monitoring.

These results are consistent with prior research documenting esketamine’s rapid onset, attributed to its NMDA receptor antagonism and downstream neuroplastic effects (Murrough et al., 2013; Stapper et al., 2025; Zarate et al., 2006). Retrospective outcomes indicate that intranasal esketamine can induce notable symptom improvements within the first few weeks of therapy with response and remission rates substantially rising by month three (Martinotti et al., 2022; Popova et al., 2019). In contrast, past observational clinical reports of rTMS indicate benefits typically emerge after several weeks, commonly in the second to fourth week, with some patients only responding after the full 4–6 week acute course (Prodi et al., 2024). rTMS is theorized to act via longer-term modulation of neural circuitry, following a more gradual trajectory (O’Reardon et al., 2007). Real-world time-to-response is also shaped by differences in treatment delivery and time-in-care: esketamine concentrates prolonged supervised clinic contact into fewer visits, whereas rTMS is delivered as brief, high-frequency sessions with effects that may accumulate across repeated exposures. Accordingly, earlier separation may reflect both faster symptom changes and earlier opportunities for assessment and documentation embedded in visit cadence. Observed differences in time-to-response may partially reflect differences in treatment density and symptom ascertainment embedded in standard rTMS versus esketamine visit schedules, underscoring the need for comparisons that harmonize assessment timing and include accelerated rTMS protocols. Here, comparative samples reinforce esketamine’s faster relief in TRD, while rTMS gains accumulate more gradually. This early advantage should be interpreted alongside longer-term treatment burden. In clinical practice, many patients who respond to esketamine continue weekly or every-other-week maintenance treatment to reduce relapse risk, whereas continuation or maintenance rTMS is less standardized (Łysik et al., 2025) (Fig. 1). Although esketamine may produce faster acute improvement, the time and logistical demands of ongoing maintenance may exceed those of rTMS and should factor into treatment selection.

This extended to the treatment of SI, a critical outcome in TRD. Although SI is embedded in the PHQ-9 total score, the SI item was isolated in a separate analysis to capture the unique trajectory of suicidality, an outcome with distinct clinical urgency, rather than broader symptom change. Median time-to-SI-improvement was significantly shorter for esketamine (9 days) than rTMS (26 days), indicating earlier observed SI improvement. This aligns with esketamine’s current FDA approval for acute suicidality and supports its use in clinical scenarios requiring urgent symptom alleviation (Canuso et al., 2019). As SI outcomes in past pivotal trials did not clearly separate from placebo (Fu et al., 2020)(Ionescu et al., 2021), nonspecific factors (e.g., intensive monitoring/clinical contact) may contribute, especially given the longer in-clinic time for esketamine (~2 h) versus rTMS (minutes).

Recent studies have explored differential efficacy and response trajectories of rTMS and esketamine for TRD. In a retrospective naturalistic study (*n* = 24), ketamine produced faster responses than rTMS, particularly in severely ill patients, aligning with the current findings (Mikellides et al., 2022). Similarly, Al-Sabbagh et al. (2022) examined esketamine following rTMS (*n* = 13), suggesting that sequential use of therapies may improve outcomes, particularly for patients who respond inadequately to rTMS alone. In contrast, recent real-world studies comparing accelerated rTMS with esketamine have found higher early response rates for rTMS at one month, with outcomes converging over longer follow-up (Pettorruso et al., 2023; Prato et al., 2026). Such variability may reflect not only treatment modality, but also surrounding procedural and clinical factors, including protocol intensity, accelerated versus standard rTMS delivery, and the degree of monitoring embedded in hospital-based care. This suggests treatment efficacy and speed-of-response may be protocol dependent, particularly when accelerated rather than standard rTMS. Observed response rates in the current study align with meta-analytic estimates of 49–55% in controlled rTMS studies (Brunoni et al., 2017), and the 68.82% closely matches the 64–70% range reported in Phase III esketamine trials (Canuso et al., 2019). This concordance suggests that these naturalistic findings largely recapitulate efficacy seen under trial conditions.

Exploratory analyses suggested that responses were significantly modulated by clinical features: comorbid anxiety disorders were associated with a lower likelihood of response, consistent with evidence that anxiety is a strong negative predictor of rTMS efficacy (Wang et al., 2021), possibly due to heightened limbic reactivity (George et al., 2013). Additionally, benzodiazepine use predicted poorer rTMS response, plausibly reflecting its known dampening effects on neuroplasticity which may counteract the excitatory intent of rTMS protocols (Hunter et al., 2019). Interestingly, former tobacco use was associated with improved rTMS outcomes, consistent with previous findings on a smaller subset of this rTMS database (Benster et al., 2025) and possibly reflecting underlying neurobiological differences related to dopaminergic tone or neuroplasticity.

In contrast, no predictors reached significance for esketamine. However, given the smaller esketamine sample size, these null findings should be interpreted cautiously and may reflect limited statistical power rather than the absence of clinically meaningful moderators.

This investigation has several limitations including non-randomized real-world data that may introduce selection biases, and a small subset who received both treatments. Additionally, TRD status was based on EMR-documented antidepressant nonresponse, and details regarding dose adequacy, treatment duration, and adherence were not consistently available; therefore, the TRD definition may reflect real-world clinical complexity rather than a standardized operational definition. To confirm results were not driven by this subset, a sensitivity analysis excluding these patients (*n* = 22) showed essentially unchanged curves (Supplemental Fig. 4), supporting robustness to treatment-history overlap. Treatment protocols were not fully standardized, and the rTMS group pooled multiple clinical protocols, including iTBS, bilateral TBS, high-frequency left DLPFC, and low-frequency right DLPFC stimulation; this heterogeneity may introduce variability and obscure protocol-specific response trajectories. In routine care, rTMS was typically delivered as brief weekday sessions with PHQ-9 assessments administered intermittently (on average once per week), whereas esketamine involved fewer, but longer, supervised visits with assessments collected at each session. Differences in visit structure, monitoring intensity, and naturalistic follow-up may have favored earlier detection of response in the esketamine group, although harmonized 7-day and 14-day assessment-window sensitivity analyses yielded consistent findings. The longer median protocol duration in the esketamine group may also have allowed greater accumulation of response and remission events. Although minimal session thresholds were applied to define treatment initiation, dropout or tolerability differences between modalities could influence trajectories. Right-censoring mitigates, but does not fully eliminate this limitation. Because response was the primary time-to-event endpoint, remission was summarized descriptively rather than analyzed as a parallel longitudinal outcome. Lastly, unequal sample sizes may have constrained subgroup analyses, particularly for esketamine moderators. SI improvement was defined as a ≥ 1-point reduction in PHQ-9 item 9, which may be sensitive to change but less specific for clinically meaningful improvement than dedicated suicide severity measures. Limitations notwithstanding, these findings contribute insights into esketamine and rTMS performance in clinical practice, particularly in response speed and clinical modulators. Prospective double-blind studies are needed to replicate these results, ideally with harmonized protocols and randomization to clarify the extent to which observed differences reflect treatment effects versus care structure. Additional predictors, especially neurophysiological, genetic, or cognitive biomarkers, could greatly enhance individualized tailoring of treatments.

## 5. Conclusion

This real-world comparison suggests esketamine produces a faster time-to-response than rTMS, particularly for SI improvement, making it a compelling option for patients in acute distress. rTMS, while effective, demonstrated a more gradual response, with outcomes significantly influenced by baseline factors: anxiety comorbidity, benzodiazepine use, and former tobacco use. These findings may help inform individualized clinical discussions about expected response trajectories in TRD, while prospective randomized studies are needed before these results can be used to guide stratified treatment selection. Esketamine may be beneficial for those requiring rapid symptom relief, whereas rTMS remains an effective option, particularly for patients for whom esketamine is contraindicated, burdensome, or less clinically appropriate, while baseline factors such as anxiety comorbidity and benzodiazepine use may help inform expectations regarding response trajectory. Future studies should focus on refining treatment protocols, exploring additional predictors, and assessing the long-term effects of these interventions in broader, more diverse patient populations.

## Supplementary Material

Supplement

## Figures and Tables

**Table 1:** Baseline demographic and clinical characteristics of patient sample.

Characteristic	rTMS (n = 279)	Esketamine (n = 93)	Test Statistic	P-value
Mean Age (SD)	53.68 (17.21)	54.13 (17.19)	*t* = −0.218	0.827
Gender Distribution(M/F/O)	101/177/1	39/52/2	χ^2^ = 4.029	0.133
Mean BMI (SD)	27.16 (5.89)	27.78 (6.41)	*t* = −0.824	0.411
History of Trauma (%)	46.3%	63.8%	χ^2^ = 5.501	0.019*
Anxiety Comorbidity (%)	75.4%	78.7%	χ^2^ = 0.150	0.699
Concurrent Substances (%)	55.4%	53.7%	χ^2^ = 0.010	0.921
Former Tobacco Use(%)	15.7%	7.4%	χ^2^ = 2.608	0.106
Mean Baseline PHQ-9 (SD)	17.61 (5.39)	17.12 (4.87)	*t* = 0.818	0.415
Previously Underwent ECT	16.8%	15.0%	χ^2^ = 0.404	0.686
Previously Underwent rTMS	11.1%	47.3%	χ^2^ = 56.79	0.001*

* Indicates statistically significant between-group difference, *p* < 0.05.

## Data Availability

The data underlying this article are not publicly available at this time. The dataset contains sensitive patient information derived from electronic medical records and is subject to institutional and privacy restrictions.

## Appendix A. Supplementary data

Supplementary data to this article can be found online at https://doi.org/10.1016/j.jad.2026.122107.
