# Supplementary material for: Comparing transcranial magnetic stimulation and esketamine treatment response trajectories in resistant depression
Source: J Affect Disord. Author manuscript; Available in PMC 2026 Jul 26. (PMC13401942; doi:10.1016/j.jad.2026.122107)
Supplement: Supplement [file NIHMS2194805-supplement-Supplement.docx]

**SUPPLEMENTAL FIGURES**


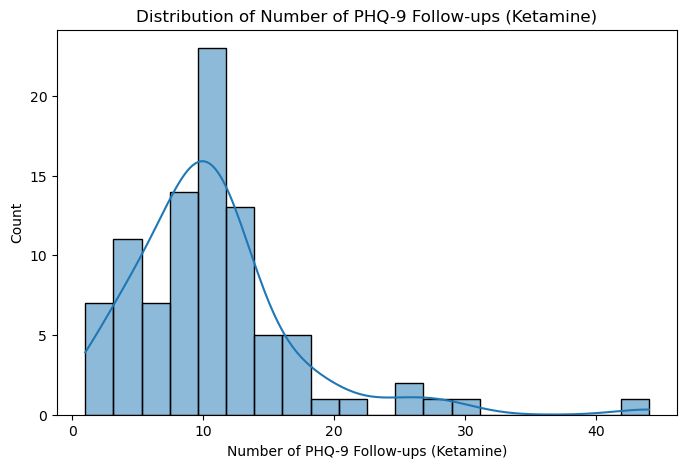

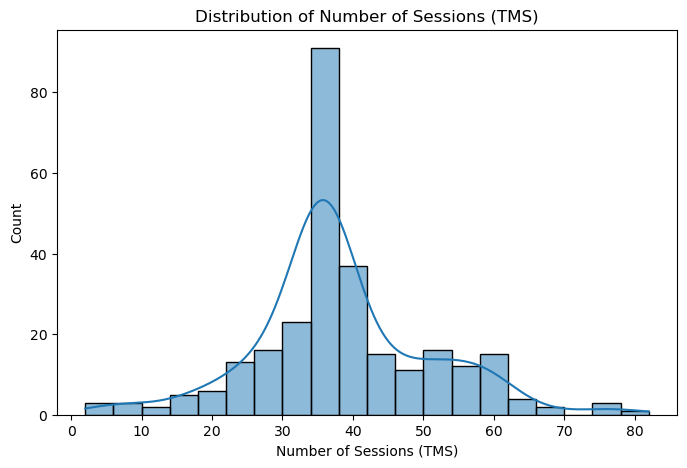


***Supplemental Figure S1****: Distribution of treatment exposure for esketamine and rTMS.* ***Left****: Number of follow-ups obtained during esketamine treatment (PHQ-9 were assessed at each follow-up for esketamine and at varying intervals for rTMS).* ***Right****: number of rTMS treatment sessions completed per patient.*


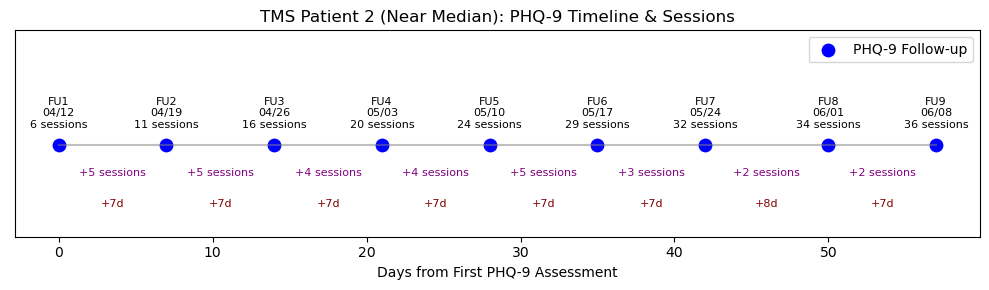


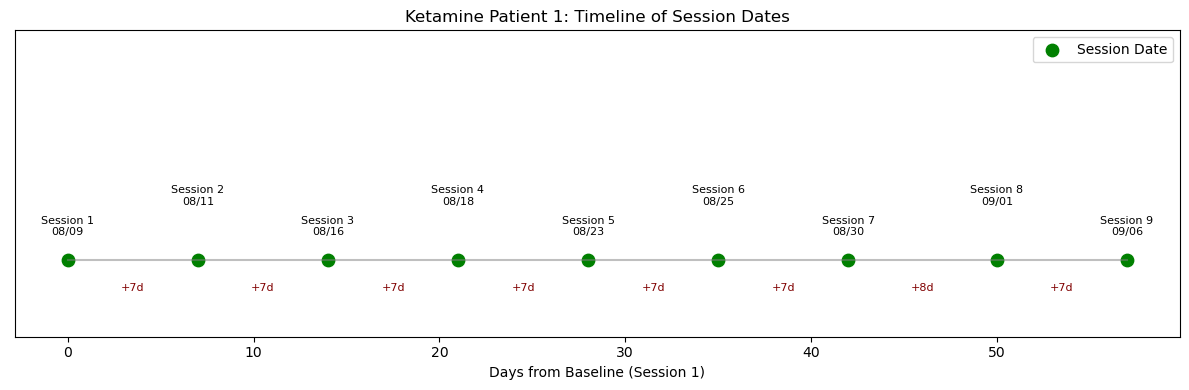


***Supplemental Figure S2****: example patient treatment spacing.* ***Top****: An example rTMS patient illustrating that PHQ-9 assessments occurred intermittently, often every 5–7 sessions. Because rTMS sessions are delivered multiple times per week, the number of sessions between PHQ-9 timepoints varies, and PHQ-9s do not occur at every treatment encounter.* ***Bottom:*** *An example esketamine patient illustrating that PHQ-9 assessments were typically completed at each treatment session, resulting in a 1:1 correspondence between sessions and PHQ-9 follow-ups. This difference in spacing demonstrates why rTMS has many more total treatment sessions than PHQ-9 observations, whereas esketamine shows a closer match between the number of sessions and the number of PHQ-9 follow-ups.*


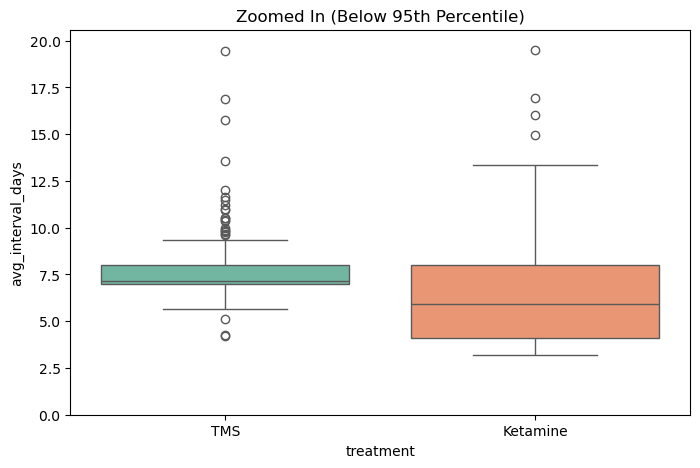


***Supplemental Figure S3****: treatment spacing for rTMS (green) and esketamine (orange).*

| **Predictor** | **rTMS HR (95% CI)** | **rTMS p-value** | **Esketamine HR (95% CI)** | **Esketamine p-value** |
| --- | --- | --- | --- | --- |
| Age | 1.01 (1.00-1.02) | 0.125 | 1.00 (0.98-1.02) | 0.956 |
| Anxiety Disorder Comorbidity | 0.69 (0.49-0.98) | **0.039*** | 0.88 (0.45-1.74) | 0.716 |
| BMI | 0.95 (0.80-1.13) | 0.595 | 0.97 (0.70-1.33) | 0.841 |
| Former Tobacco Use | 1.31 (1.08-1.59) | **0.0059*** | 1.30 (0.85-1.97) | 0.221 |
| Benzodiazepine Use | 0.73 (0.53-0.99) | **0.0459*** | 1.19 (0.68-2.09) | 0.532 |
| Trauma History | 0.84 (0.61-1.15) | 0.3006 | 0.70 (0.40-1.23) | 0.217 |
| Session Number | 0.99 (0.97-1.00) | 0.154 | 1.02 (0.98-1.06) | 0.28 |

***Supplementary Table S1. Results of Cox Regression.*** *Hazard ratios (HRs), 95% confidence intervals, and p-values are shown separately for rTMS and esketamine. Each model estimates the association between baseline clinical and demographic variables and the instantaneous likelihood of achieving a ≥50% reduction in PHQ-9 symptoms within that treatment modality. Significant predictors (p<0.05) appear only in the rTMS model, whereas no covariates reached significance for esketamine.*

**Supplementary Table S2. Kaplan-Meier Estimates for Time to SI Improvement**

| **Time Interval** | **rTMS Survival Probability (No Response)** | **Esketamine Survival Probability (No Response)** |
| --- | --- | --- |
| 5 days | 0.903 | 0.6 |
| 10 days | 0.77 | 0.471 |
| 20 days | 0.605 | 0.339 |
| 30 days | 0.416 | 0.26 |
| 60 days | 0.325 | 0.184 |
| 90 days | 0.17 | 0.153 |
| 120 days | 0.17 | 0.153 |
| 150 days | 0.17 | 0.153 |
| 180 days | 0.17 | 0.153 |
| Median Time to Response | 26 | 9 |
| Log-Rank Test Statistic | 10.681 | 0.001082 |

**Supplemental Table S3. Treatment Exposure and Overall Outcome Metrics by Modality**

| **Measure** | **rTMS (n=279)** | **Esketamine (n=93)** |
| --- | --- | --- |
| Mean # of Sessions | 38.45 | 10.6 |
| Median # of Sessions | 36 | 10 |
| Session Range | 5-82 | 3-44 |
| Response (at any time) Rate | 59.43% | 68.82% |
| Remission (at any time) Rate | 40.14% | 45.16% |

**Supplementary Table S4. Logistic Regression Results**

| **Predictor** | **rTMS Coef** | **rTMS Std Err** | **rTMS z** | **rTMS p** | **Ket Coef** | **Ket Std Err** | **Ket z** | **Ket p** |
| --- | --- | --- | --- | --- | --- | --- | --- | --- |
| Age | 0.0095 | 0.009 | 1.106 | 0.269 | 6.42E-05 | 0.015 | 0.004 | 0.997 |
| Trauma Hx | 0.2317 | 0.263 | 0.88 | 0.379 | -0.4783 | 0.508 | -0.942 | 0.346 |
| Benzodiazepines | -0.7053 | 0.294 | -2.401 | **0.016*** | 0.6621 | 0.512 | 1.224 | 0.221 |
| Substance Use | 0.4135 | 0.174 | 2.377 | **0.017*** | 0.1652 | 0.489 | 0.338 | 0.625 |
| BMI | -0.1228 | 0.13 | -0.946 | 0.344 | 0.0825 | 0.226 | 0.365 | 0.715 |
| Num of Comorbidities | -0.1121 | 0.114 | -0.981 | 0.327 | -0.2058 | 0.179 | -1.15 | 0.25 |
| Baseline PHQ-9 | 0.0737 | 0.102 | 0.722 | 0.47 | 0.2156 | 0.12 | 1.803 | 0.071 |


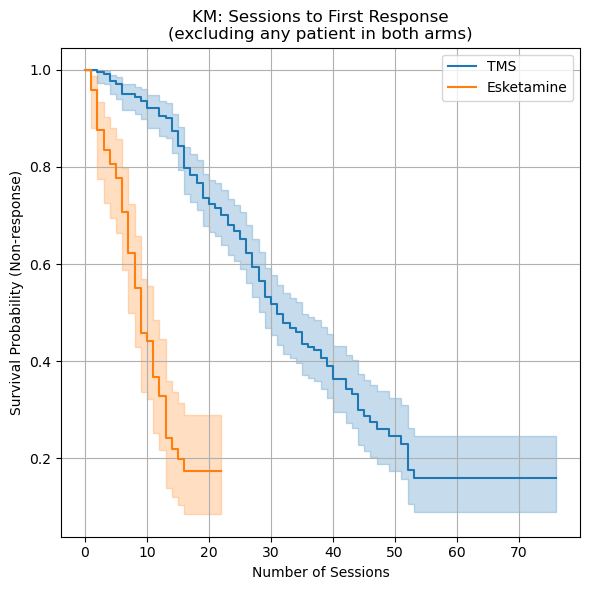


***Supplemental Figure S4****: KM survival curves for number of treatment sessions to first clinical response in rTMS (blue) versus esketamine (orange), after excluding all patients who had received both modalities. Shaded regions denote 95% confidence intervals. The overall pattern and statistical separation between the two arms remain very similar to the primary analysis.*

***Supplemental Table S5: Predictor Selection and Rationale***

| **Category** | **Predictor** | **Rationale** |
| --- | --- | --- |
| **Demographics** | Age | Neuroplasticity tends to decline with age, which may affect both rTMS and ketamine response. Older age has been associated with slower or reduced response to rTMS and may influence ketamine metabolism.(28) |
| **Clinical History** | History of Trauma | Trauma exposure is linked to altered fronto-limbic connectivity and stress-response systems, which may interact with neuromodulatory effects of rTMS and ketamine.(29) |
| **Diagnosis** | Anxiety Comorbidity | Comorbid anxiety disorders have been shown to reduce the efficacy of rTMS in MDD, possibly due to differences in network responsivity or symptom interference.(30) |
| **Substance Use** | Former Tobacco Use | Previous findings from this dataset suggested former tobacco use was associated with better rTMS response, potentially due to upregulation of dopaminergic tone and altered cortical excitability.(11) |
| **Medication** | Benzodiazepines | Benzodiazepines can suppress cortical excitability and interfere with neuroplastic mechanisms, potentially reducing response to rTMS and blunting ketamine efficacy.(31) |
| **Physical Health** | BMI | Higher BMI may alter drug pharmacokinetics and neuromodulation response, particularly for ketamine due to its lipophilic properties. rTMS response has also shown mixed associations with BMI.(32) |

***Supplementary Table S6 — Schoenfeld residuals test (proportional hazards)***

| **Variable** | **Chi-square** | **df** | **p-value** |
| --- | --- | --- | --- |
| age_years | 1.1335 | 1 | 0.2870 |
| anxiety | 0.0740 | 1 | 0.7856 |
| baseline_phq | 2.3085 | 1 | 0.1287 |
| benzo | 3.5026 | 1 | 0.0613 |
| bmi | 2.1508 | 1 | 0.1425 |
| former_tobacco | 0.2013 | 1 | 0.6537 |
| iptw_wt | 0.3878 | 1 | 0.5334 |
| trauma | 2.4777 | 1 | 0.1155 |
| treatment | 4.7745 | 1 | **0.0289*** |
| GLOBAL | 8.6467 | 9 | 0.4705 |

*Schoenfeld residuals test for the proportional hazards assumption (variable-wise chi-square, degrees of freedom, and p-values). Tests were computed from the fitted Cox model using Schoenfeld residuals (lifelines proportional_hazard_test, time_transform='rank'). A small p-value (<0.05) indicates potential departure from the proportional hazards assumption; here the treatment covariate shows a significant variable-wise result (p = 0.0289). Variable-wise p-values are unadjusted for multiple testing.*

***Supplementary Methods S1. Penalized Cox sensitivity analysis***

*As a sensitivity analysis, an unweighted, covariate-adjusted Cox proportional hazards model was fit for time to clinical response. The model included treatment (esketamine vs rTMS) and the same baseline covariates used in the IPTW analysis: age, baseline PHQ-9, BMI, anxiety-disorder comorbidity, psychological-trauma history, and former tobacco use.*

*Hyperparameters were tuned with 5-fold cross-validation using GridSearchCV over the inverse-regularization parameter C and the elastic-net mixing parameter (l1_ratio, corresponding to α). Candidate regularization strengths were λ ∈ {0.01, 0.10, 1.0}, where λ = 1/C. Convergence diagnostics indicated that a ridge solution (l1_ratio = 0) with λ = 0.10 provided the best fit (cross-validated AUC = 0.61). In this penalized Cox model, esketamine was associated with a 23% higher hazard of response relative to rTMS (HR = 1.23, 95% CI 1.04–1.45, p = 0.014), consistent with the primary IPTW estimate (HR = 1.62, 95% CI 1.16–2.26, p = 0.005). Full coefficient estimates and confidence intervals are provided in Supplementary Table S7.*

***Supplementary Table S7****. Covariate-adjusted penalized Cox proportional-hazards model for time to clinical response.*

| **Variable** | **Hazard ratio** | **95 % CI** | ***p*** |
| --- | --- | --- | --- |
| Esketamine vs rTMS | **1.23** | 1.04–1.45 | **0.014*** |
| Age (per SD) | 1.06 | 0.99–1.14 | 0.12 |
| Baseline PHQ-9 (per SD) | 0.98 | 0.90–1.05 | 0.54 |
| BMI (per SD) | 0.97 | 0.90–1.03 | 0.31 |
| Anxiety comorbidity (yes) | **0.80** | 0.67–0.95 | **0.009*** |
| Trauma history (yes) | 0.92 | 0.80–1.07 | 0.29 |
| Former tobacco use (yes) | **1.27** | 1.05–1.55 | **0.017*** |

Model concordance was 0.61, partial AIC 2262.5, and the likelihood-ratio test for the full model was χ²(7) = 12.2 (*p* = 0.095). Covariate-adjusted penalized Cox proportional-hazards model for time to clinical response (penalized Cox; ridge penalty).

*Esketamine treatment was independently associated with a 23% increased hazard of achieving a 50% PHQ-9 reduction relative to rTMS, translating to a covariate-adjusted median time-to-response of 37 days for esketamine and 46 days for rTMS. The presence of a comorbid anxiety disorder was linked to slower improvement (20 % reduction in hazard), whereas former-tobacco use predicted faster response (27 % increase in hazard). Age, baseline depressive-symptom severity, BMI and trauma history were not significant predictors in the adjusted model.*

***Supplementary Table S8*.** *Covariate balance before and after inverse probability of treatment weighting*

| **Covariate** | **Unweighted SMD** | **Weighted SMD** |
| --- | --- | --- |
| Age | 0.007 | 0.013 |
| Baseline PHQ-9 | 0.073 | 0.026 |
| BMI | 0.126 | 0.007 |
| Anxiety comorbidity | 0.112 | 0.013 |
| Trauma history | 0.381 | 0.032 |
| Former tobacco use | 0.266 | 0.014 |
| **Maximum absolute SMD** | **0.381** | **0.032** |
| Covariates with absolute SMD > 0.10 | 4/6 | 0/6 |

*Stabilized inverse probability of treatment weights were Winsorized at the 1st and 99th percentiles. Winsorized stabilized weights ranged from 0.573 to 2.089 (mean = 0.992, SD = 0.223; median = 0.951), and the effective sample size after weighting was 353. SMD = standardized mean difference; IPTW = inverse probability of treatment weighting.*
